# Ocean acidification reduces induction of coral settlement by crustose coralline algae

**DOI:** 10.1111/gcb.12008

**Published:** 2012-09-25

**Authors:** Nicole S Webster, Sven Uthicke, Emanuelle S Botté, Florita Flores, Andrew P Negri

**Affiliations:** Australian Institute of Marine SciencePMB 3, Townsville Mail Centre, Townsville, Qld 4810 Australia

**Keywords:** climate change, crustose coralline algae, microorganism, ocean acidification, symbiosis

## Abstract

Crustose coralline algae (CCA) are a critical component of coral reefs as they accrete carbonate for reef structure and act as settlement substrata for many invertebrates including corals. CCA host a diversity of microorganisms that can also play a role in coral settlement and metamorphosis processes. Although the sensitivity of CCA to ocean acidification (OA) is well established, the response of their associated microbial communities to reduced pH and increased CO_2_ was previously not known. Here we investigate the sensitivity of CCA-associated microbial biofilms to OA and determine whether or not OA adversely affects the ability of CCA to induce coral larval metamorphosis. We experimentally exposed the CCA *Hydrolithon onkodes* to four pH/*p*CO_2_ conditions consistent with current IPCC predictions for the next few centuries (pH: 8.1, 7.9, 7.7, 7.5, *p*CO_2_: 464, 822, 1187, 1638 μatm). Settlement and metamorphosis of coral larvae was reduced on CCA pre-exposed to pH 7.7 (*p*CO_2_ = 1187 μatm) and below over a 6-week period. Additional experiments demonstrated that low pH treatments did not directly affect the ability of larvae to settle, but instead most likely altered the biochemistry of the CCA or its microbial associates. Detailed microbial community analysis of the CCA revealed diverse bacterial assemblages that altered significantly between pH 8.1 (*p*CO_2_ = 464 μatm) and pH 7.9 (*p*CO_2_ = 822 μatm) with this trend continuing at lower pH/higher *p*CO_2_ treatments. The shift in microbial community composition primarily comprised changes in the abundance of the dominant microbes between the different pH treatments and the appearance of new (but rare) microbes at pH 7.5. Microbial shifts and the concomitant reduced ability of CCA to induce coral settlement under OA conditions projected to occur by 2100 is a significant concern for the development, maintenance and recovery of reefs globally.

## Introduction

Atmospheric carbon dioxide (CO_2_) concentrations have increased over the past 250 years from a partial pressure (*p*CO_2_) of 280 μatm to a present level of nearly 400 μatm (IPCC, 2007). The *p*CO_2_ is projected to increase further to between 730 and 1088 μatm by 2100 depending on the model type and assumptions applied (Meehl *et al*., 2007). Uptake of CO_2_ by the ocean alters the carbonate chemistry of seawater resulting in a reduction in pH and carbonate saturation and increased dissolved inorganic carbon availability (Caldeira & Wickett, 2003; Raven *et al*., 2005), which can directly affect organisms which accrete carbonate as part of their physical structure. Experiments have demonstrated that increasing *p*CO_2_ causes reduced calcification of reef-building corals (Schneider & Erez, 2006; Anthony *et al*., 2008) and crustose coralline algae (Anthony *et al*., 2008; Jokiel *et al*., 2008), supporting the hypothesis that a continuation of this rapid increase in ocean acidification (OA) poses a long-term threat to the viability of coral reefs globally (Hoegh-Guldberg *et al*., 2007).

Although the primary focus of OA impacts on marine invertebrates has been on calcification, OA has been shown to negatively affect reproduction and early developmental stages in calcifying marine invertebrates (Byrne, 2011) including corals (Albright, 2011). The reproductive and recruitment success of corals is critical to the maintenance of coral reef ecosystems and their recovery after disturbances such as bleaching (Ritson-Williams *et al*., 2009). Some processes such as gametogenesis (Fine & Tchernov, 2007; Jokiel *et al*., 2008), larval survival (Albright *et al*., 2008; Suwa *et al*., 2010; Anlauf *et al*., 2011) and settlement (Albright *et al*., 2008; Anlauf *et al*., 2011) seem unaffected by OA; however, increased *p*CO_2_ (low pH) has been demonstrated to reduce fertilization success (Albright *et al*., 2010) and settlement of coral larvae indirectly by modifying the chemistry or community composition of their preferred settlement substratum (Albright *et al*., 2010; Albright & Langdon, 2011; Doropoulos *et al*., 2012).

Crustose coralline algae (CCA) are a critical structural component of all coral reefs and the carbonate it accretes contains a greater proportion of magnesium-calcite than coral. As a consequence, CCA is more susceptible than coral to the reduced carbonate saturation state as *p*CO_2_ increases (Morse *et al*., 2006) and multiple experiments have documented this vulnerability (Anthony *et al*., 2008; Kuffner *et al*., 2008; Martin & Gattuso, 2009; Diaz-Pulido *et al*., 2012). The impacts of OA on CCA may have downstream effects on coral recruitment as diverse genera of Indo-Pacific and Caribbean hard and soft coral larvae (*Acropora, Agaricia*,*Alcyonium, Cyphastraea*,*Leptoseris*,*Favia*,*Fungia*,*Goniastrea*,*Symphyllia*) metamorphose in response to CCA and/or associated biofilms (Sebens, 1983; Morse *et al*., 1996; Negri *et al*., 2001; Heyward & Negri, 2010). CCA also represents an important permanent attachment substratum for coral recruits (Harrington *et al*., 2004) and settlement on other natural and artificial substrata is likely to be mediated by associated microbial biofilms and/or early successional CCA colonies (Webster *et al*., 2004). Three studies have recently shown that increased *p*CO_2_ affects mixed algal communities and in turn reduces coral larval settlement. Albright and colleagues exposed algae on tiles to three *p*CO_2_ treatments for 40 days and observed reduced larval settlement along with apparent reductions in CCA cover and an increased abundance of turf algae (Albright *et al*., 2010; Albright & Langdon, 2011). Doropoulos and colleagues showed that exposing mixed algal communities on tiles to elevated *p*CO_2_ for 60 days significantly reduced CCA cover and the subsequent settlement of coral larvae (Doropoulos *et al*., 2012). In that study, CCA species important for coral recruitment *Hydrolithon* spp. and *Titanaderma* spp. (Harrington *et al*., 2004) were affected to a greater extent than other algal species. Although reductions in CCA abundance were reported in all studies and may be a primary mechanism explaining reduced larval settlement, another experiment indicated that elevated *p*CO_2_ can reduce coral settlement over a short 6-days period in the absence of changes in CCA abundance (Doropoulos *et al*., 2012). Reduced settlement in the absence of changes to CCA abundance indicates that the water chemistry (increased *p*CO_2_ or reduced pH) may also directly affect the production or structure of the chemical inducer for settlement produced by CCA or its associated microbial community.

Dense bacterial biofilms occur on the surface of CCA and play important roles in inducing larval metamorphosis for numerous invertebrate species (Johnson & Sutton, 1994; Huggett *et al*., 2006). For instance, bacteria (and their metabolites) isolated from CCA (Tebben *et al*., 2011) and mixed bacterial communities developed on coral reefs can induce settlement and metamorphosis in coral larvae. Negative impacts of OA on the distribution or abundance of microbes involved in metamorphosis processes could therefore have serious implications for reef-building, maintenance and recovery. Recent studies have shown that OA affects community structure of tropical microbial biofilms with increases and decreases in specific bacterial groups correlating with *p*CO_2_ (Witt *et al*., 2011). The effects of environmental disturbance on the functional roles of microbes associated with CCA remain largely unknown, although elevated seawater temperature was recently found to change the microbial biofilms on the CCA *Neogoniolithon fosliei*, with a concomitant reduction in coral larval settlement (Webster *et al*., 2011). OA may affect recruitment by either altering microbial production of morphogenic signalling compounds or by shifting the community structure with a loss of potentially important species or the introduction of inhibitory strains. In this study, we exposed the CCA species *Hydrolithon onkodes* to elevated *p*CO_2_ conditions for 6 weeks to: (i) investigate the impact of OA on community structure of CCA-associated microbial biofilms, (ii) assess whether or not OA affects the ability of CCA to induce metamorphosis of coral larvae, and (iii) assess whether or not acute exposure of larvae to conditions of OA affects settlement and metamorphosis.

## Materials and methods

### Preparation and mounting of CCA

**Figure 1:**
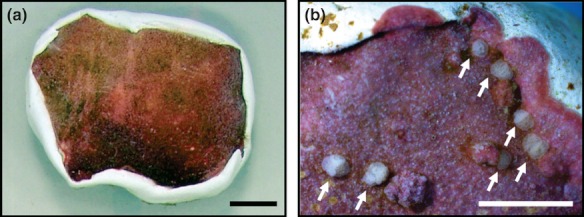
Photographs of *Hydrolithon onkodes*: (a) freshly mounted crustose coralline algae (CCA) on a glass tile with underwater epoxy to avoid exposure of dead CCA skeleton and (b) with newly settled corals on the CCA surface following exposure to pH 7.7 for 6 weeks. Note the new algal growth on the epoxy around the top right of the image. Scale bar = 0.5 cm, arrows indicate newly settled recruits.

The CCA *Hydrolithon onkodes* (formerly *Porolithon onkodes*) is a dominant shallow water species of mid-shelf reefs of the Great Barrier Reef (GBR) (Harrington, 2004) and has been demonstrated to induce the settlement and metamorphosis of *Acropora* coral larvae (Heyward & Negri, 1999; Harrington *et al*., 2004). Over 50 pieces of *H. onkodes* were collected at Davies Reef, GBR (18°50.558′S, 147°37.618′E) from the reef tops and crests at 2–5 m depths. CCA were maintained in outdoor aquaria at the Australian Institute of Marine Science (AIMS) Townsville, Australia, with flow-through filtered seawater (5 μm) under 70% shading (maximum 350 μmol photons m^−2^ s^−1^) and ambient temperature (26–28 °C) for 7 days. The CCA pieces were further fragmented into random 30 × 20 mm sections and these were mounted onto clear glass tiles with the minimum amount of underwater epoxy (Sellys Aqua Knead-It) so that only the live CCA was exposed to seawater (Fig 1a). Mounting the CCA for these types of experiments is critical to eliminate the potential influence of elevated *p*CO_2_ on unprotected dead CCA skeleton and potential for dead skeleton to induce settlement. Larval settlement has previously been described on dead CCA (Heyward & Negri, 1999) and may result from chemical inducers that had been produced by the live CCA or associated biofilms. The present study was only concerned with live CCA because it cannot be ascertained that the dead skeleton was laid down by the same species. The mounted CCA was allowed to acclimate in the indoor aquarium for a week prior to acidification exposures.

### Coral collection and larval cultivation

Five colonies each of the corals *Acropora millepora* and *Acropora tenuis* (30–50 cm diameter) were selected haphazardly from a depth between 1–3 m at Pelorus Island, GBR (18°33.487′S, 146°30.198′E) and transported to the AIMS outdoor aquarium facility described above. The CCA and coral species used in the present study are present at both the CCA and coral collection sites. The gametes from all five colonies of each species were collected following synchronous spawning on November 23, 2010 and the azooxanthellate larvae of each species cultured separately in indoor flow-through aquaria (26–28 °C) using methods described in (Negri & Heyward, 2000).

### Experimental design

Mounted CCA were exposed to four seawater *p*CO_2_ conditions (Table 1) for 6 weeks to allow sufficient time for biofilm succession in response to altered *p*CO2 conditions. The experiments were conducted in an indoor flow-through computer-controlled CO_2_ aquarium dosing system (Uthicke *et al*., 2012). Seawater for each treatment was filtered to 0.5 μm and acidified in 450 l header tanks using a CO_2_ gas injection system (AquaMedic, Bissendorf, Germany), regulated by feedback from pH sensors in the header tanks (±0.01 pH unit). All sensors were calibrated with NIST buffers. Target pHs for the elevated *p*CO2 concentrations were set at pH_NIST_ 7.90, 7.70 and 7.50 ± 0.05, yielding *p*CO_2_ conditions ranging from the aquarium system control of 464 μatm (pH 8.1) to 1638 (pH 7.5) μatm projected by 2100 and beyond (IPCC, 2007; Moss *et al*., 2010). Water from the header tanks was pumped into three replicate 17.5 l treatment tanks, with flow-through conditions ensuring that water was replaced every 45 min. Water movement within the treatment tanks was maintained with small submerged pumps and the algae was maintained under a 12:12 hours diurnal cycle at 180–200 μmol photons m^−2^ s^−1^ (55 W, 10 000 K compact fluorescent tubes) and 26.5 ± 1 °C.

**Table 1:** Average water chemistry in treatment tanks during experiments. pH (Total) represents the pH value as calculated from measured Alkalinity (A_T_) and dissolved inorganic carbon measurements (C_I_). pH [NIST] are the mean of measurements taken over 30 days. Values in brackets represent 95% asymmetrical confidence intervals for *N* = 3 sampling periods or *N* = 30 pH [NIST]. Values in brackets given for other parameters represent SD for *N* = 3 periods

Treatment[Target]	pH [NIST]	pH [Total]	Temp [°C]	A_T_ [μmol kg^−1^ SW]	C_I_ [μmol kg^−1^ SW]	*p*CO_2_ [μatm]	Ω_Ca_	Ω_Ar_
8.1	8.12 (8.06–8.19)	7.98 (7.98–7.99)	27.0 (0.5)	2255 (38)	1981 (29)	464 (7)	4.75 (0.18)	3.15 (0.12)
7.9	7.92 (7.88–7.96)	7.77 (7.75–7.80)	27.0 (0.6)	2260 (37)	2090 (23)	822 (42)	3.20 (0.24)	2.12 (0.16)
7.7	7.78 (7.74–8.84)	7.63 (7.58–7.69)	27.1 (0.5)	2259 (37)	2145 (41)	1187 (171)	2.44 (0.33)	1.61 (0.22)
7.5	7.61 (7.57–7.65)	7.51 (7.43–7.59)	27.3 (0.8)	2263 (34)	2193 (42)	1638 (324)	1.90 (0.35)	1.26 (0.24)

Water (250 ml) from each treatment was sampled over three periods for total alkalinity (A_T_) and dissolved inorganic carbon (C_I_) in each treatment tank (Table 1). Alkalinity and C_I_ were determined as previously described (Uthicke *et al*., 2012). Additional pH readings were conducted in each treatment tank 30 times throughout the experiment using a potentiometric pH probe (console: OAKTON, Vernon Hills, IL, USA; pH probe: EUTECH, Vernon Hills, IL, USA) on the NIST scale. However, daily recording of pH, temperature and mV of the aquaria and Dickson's seawater standard (Batch No. 5) allowed conversion of NIST readings to total scale for completeness. *p*CO_2_, bicarbonate, carbonate, calcite and aragonite saturation state (Ω) were calculated from A_T_ and C_I_ measurements (not pH_NIST_) using CO_2_ SYS (Table 1). The target pHs are referred to throughout the text.

### Experiments 1 and 2: effects of acidification on larval metamorphosis in the presence of CCA

In Experiment 1, we tested the effects of exposing CCA to acidified seawater for 6 weeks on larval metamorphosis under the acidified conditions (Table 2). Six mounted *H. onkodes* pieces from three replicate tanks under the four *p*CO_2_ conditions were transferred into individual 400 ml polystyrene jars under the same four *p*CO_2_ conditions. Eight-day old *A. millepora* larvae (31–35 per treatment replicate) were transferred into each jar, which was then sealed with no airspace (to eliminate gas exchange) at 26 ± 1 °C. Early settlement and metamorphosis was assessed after 18 h (Heyward & Negri, 1999), a duration long enough to establish significant levels of settlement but short enough to minimize stress on the larvae and water chemistry changes in the jars. Controls for each treatment were prepared using epoxy mounts on glass tiles without CCA and these were used in assays and assessed in the same way. The pH readings were taken in each container at the end of this period and had decreased by less than 0.1 pH units.

**Table 2:** Conditions for each experiment and treatment

Experiment	CCA treatment	Larval pre-treatment	Larval settlement (18 h)
1	pH 8.1, 6 weeks	None	pH 8.1
	pH 7.9, 6 weeks	None	pH 7.9
	pH 7.7, 6 weeks	None	pH 7.7
	pH 7.5, 6 weeks	None	pH 7.5
2	pH 8.1, 6 weeks	None	pH 8.1
	pH 8.1, 6 weeks	None	pH 7.9
	pH 8.1, 6 weeks	None	pH 7.7
	pH 8.1, 6 weeks	None	pH 7.5
3	No CCA*	pH 8.1, 24 h	pH 8.1
	No CCA*	pH 7.9, 24 h	pH 7.9
	No CCA*	pH 7.7, 24 h	pH 7.7
	No CCA*	pH 7.5, 24 h	pH 7.5

* CCA was not used in Experiment 3, instead the larvae were induced to settle with CCA extract.

In Experiment 2, we tested the effects of exposing CCA to acidified seawater during the larval metamorphosis period (31–39 larvae over an 18 h period) (Table 2). In this case the mounted *H. onkodes* pieces were maintained under control conditions (pH 8.1) for the 6-week period. These were transferred into 400 ml polystyrene jars under the four *p*CO_2_ conditions and larval metamorphosis assessed as above.

### Experiment 3: effects of acidification on larval metamorphosis in the absence of CCA

The larvae of many *Acropora* species do not undergo settlement and metamorphosis without exposure to a settlement cue from CCA or microbial biofilms (Heyward & Negri, 1999; Webster *et al*., 2004). Extracts of several species of CCA can be used to induce larval settlement in *Acropora* larvae (Heyward & Negri, 1999) and these extracts have been used to test the effects of thermal stress and pollution on coral metamorphosis in the absence of CCA (Negri & Hoogenboom, 2011; Negri *et al*., 2011). Here we examined the effects of acidification on larval metamorphosis in the absence of CCA by pre-exposing *A. millepora* and *A. tenuis* larvae to each of the pCO_2_ conditions for 24 h then initiating metamorphosis with CCA extract. This extract was prepared by extracting 4 g crushed *H. onkodes* with methanol as per (Heyward & Negri, 1999).

Eight-day old *A. millepora* and *A. tenuis* larvae (15–20) were exposed to each of the *p*CO_2_ treatments for 24 h in sealed 75 ml polystyrene containers with no airspace at 26 ± 1 °C (*n* = 6 containers per treatment) (Table 2). Larvae were then transferred to fresh treatment water containing 30 μl CCA extract, the volume of CCA extract that induces sub-maximal metamorphosis (pilot experiments indicated that 30 μl induced 79–83% metamorphosis in both species and the extract solvent was allowed to evaporate in the containers before adding seawater and larvae). Early settlement and metamorphosis was assessed after a further 18 h. Controls for each treatment were prepared identically, but without the addition of CCA extract. The pH readings were taken in each container at the end of this period and had decreased by less than 0.05 pH units.

### 16S rRNA gene cloning and sequencing

To compare the phylogenetic composition of CCA microbes after 6 weeks exposure to the different pH treatments, DNA was extracted from triplicate pieces of CCA per tank (i.e., 36 pieces in total) using the Power Plant DNA Isolation kit, MoBio Laboratories (Carlsbad, CA, USA) according to the manufacturer's protocol. The 16S rRNA gene from each CCA derived DNA extract was amplified by PCR with primers 63f and 1387r (Marchesi *et al*., 1998) using PCR conditions previously reported (Webster *et al*., 2011). The PCR products were subsequently combined for all replicate CCA pieces per treatment tank prior to sequencing (i.e., resulting in three replicate samples/pH treatment). Pooled PCR products were ligated into the TOPO TA cloning vector (Invitrogen, Mulgrave, Australia). Ligations were sent to Magrogen Inc. (Seoul, Korea) for sequencing of 96 clones/library using 63f as the sequencing primer. Macrogen Inc. transformed the ligations into Genehog electrocompetent cells (Invitrogen), grew the cells in 2xYT + ampicillin media, performed a standard alkaline lysis plasmid preparation and sequenced the clones with ABI Bigdye v3.1 chemistry on the AB3730xl (std 50 cm array run module). Clone sequences were submitted to Genbank under the accession numbers JQ178383-JQ179496.

### Phylogenetic analysis

Clone sequence quality was checked manually using Sequencher (Genesearch, Brisbane). Chimeric sequences were identified using the programs CHECK_CHIMERA (Maidak *et al*., 1999) and Bellerophon (Huber *et al*., 2004). Sequences were imported into the ARB software package (Ludwig *et al*., 1998), automatically aligned using FastAligner and manually edited. Phylogenetic trees were calculated with almost complete 16S rRNA (1400 bp) sequences for all close relatives of representative sequences from each operational taxonomic unit (OTU) using the neighbour-joining and Maximum Parsimony methods in ARB. Partial sequences were subsequently imported to the tree without changing branch topology using the ARB parsimony-interactive method. The robustness of inferred tree topologies was evaluated after 1000 bootstrap re-samplings of the neighbour-joining data in the Phylip program (Felsenstein, 1993).

### Data analyses

One-way anovas were performed on arcsine transformed% metamorphosis data in Experiments 1–3 and Fisher's LSD test was used to identify significantly different means at *P* < 0.05 using NCSS 2007 (Hintze, 2001). In Experiment 1, treatment tank was nested as a random factor. Homogeneity of variances was investigated via residual analyses.

Distance matrices of the microbial sequence data were generated in GreenGenes (DeSantis *et al*., 2006) using a Jukes Cantor correction and OTU assignment, rarefaction curves, diversity estimates and Venn diagrams were produced in mothur V1.18.1 using a distance of 0.03 (Schloss *et al*., 2009). Statistical analyses (weighted and unweighted Unifrac) were performed in mothur using a neighbour-joining tree constructed in ARB. Representative sequences for each OTU at 97% sequence similarity were selected and portrayed in neighbour-joining trees constructed in ARB as described above. The relative percentage of sequences falling into each OTU from each treatment were calculated and displayed on the tree branches.

A Non-metric Multidimensional Scaling (nMDS) ordination was performed on the relative abundance of each OTU using the Primer software package (Clarke & Gorley, 2006) to visually assess the influence of pH treatment on community composition.

## Results

### Experiment 1: larval settlement on CCA exposed to different OA conditions

**Figure 2:**
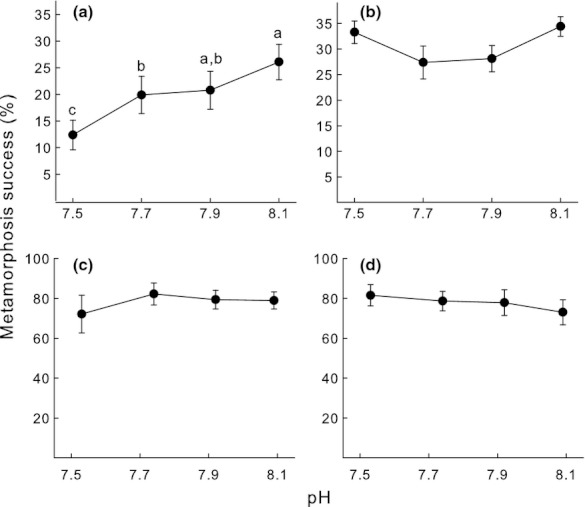
Metamorphosis success of coral larvae. (a) Experiment 1: Crustose coralline algae (CCA) pieces were pre-exposed to four treatment pH conditions for 6 weeks and coral larvae were settled at those pHs. (b) Experiment 2: CCA pieces were pre-exposed to ambient conditions and coral larvae were settled at each of the four treatment pHs. Experiment 3: (c) *Acropora millepora* and (d) *A. tenuis* larvae in response to CCA extracts following 24 h pre-exposures to different ocean acidification conditions (no solid CCA substratum present). Error bars ± SE.

The mounted CCA survived all OA conditions for 6 weeks and growth of the CCA (<10% total area) was often observed over the edges of the epoxy mounts, even in the pH 7.5 treatment (Fig 1b). After 18 h, 26 ± 3% (SE) of larvae had undergone settlement and metamorphosis in response to the CCA pieces in control (present day conditions) treatments (pH = 8.1, *p*CO_2_ = 462 μatm) (Fig 2a). Larvae settled on the CCA (Fig 1b) and also randomly on the epoxy mounts, while <2% settlement was observed on control epoxy mounts in the absence of CCA. Exposing CCA to elevated *p*CO_2_ (*p*CO_2_ = 1638 μatm/pH 7.5) for 42 days reduced the settlement success by half to 12 ± 2% of larvae (Fig 2a). Settlement success of larvae on CCA exposed to pH 7.9 and 7.7 (*p*CO_2_ = 822 and 1187 μatm) were 20 and 24% lower than the control treatments respectively. Settlement on CCA treated at 8.1 was significantly higher than on CCA treated at pHs 7.7 and 7.5, while significantly less larvae settled on the CCA treated at pH 7.5 than on CCA from all other treatments (Table S1). Metamorphosis success was significantly correlated with pCO2 (linear regression *r*^2^ = 0.94, *P* = 0.029, Fig. S1). The larvae that had not settled exhibited typical swimming and exploration behaviour and no obvious mortality was observed under these conditions.

### Experiment 2: larval settlement under different OA conditions on ambient-treated CCA

After 18 h, 33 ± 2% (SE) of larvae had settled and metamorphosed on the CCA pieces under control conditions (pH = 8.1) (Fig 2b). There was no significant difference in settlement on CCA that had been maintained under ambient conditions for 6 weeks, but settled at different pHs (anova F_(3,44)_ = 1.28, *P* = 0.29), with an overall average settlement of 31% (Fig 2b).

### Experiment 3: acute effects of OA on larval settlement in response to CCA extract

The settlement and metamorphosis success of *A. millepora* and *A. tenuis* larvae in response to CCA extract under control conditions were 79 ± 4% (SE) and 73 ± 6% respectively (Fig 2c and d). There was no effect of exposing these larvae to each of the different OA conditions for 24 h before initiating larval settlement and metamorphosis with CCA extract (anova,*A. millepora* F_(3,20)_ = 0.27, *P* = 0.85; *A. tenuis* F_(3,20)_ = 0.21, *P* = 0.89).

### Microbial community analysis

**Figure 3:**
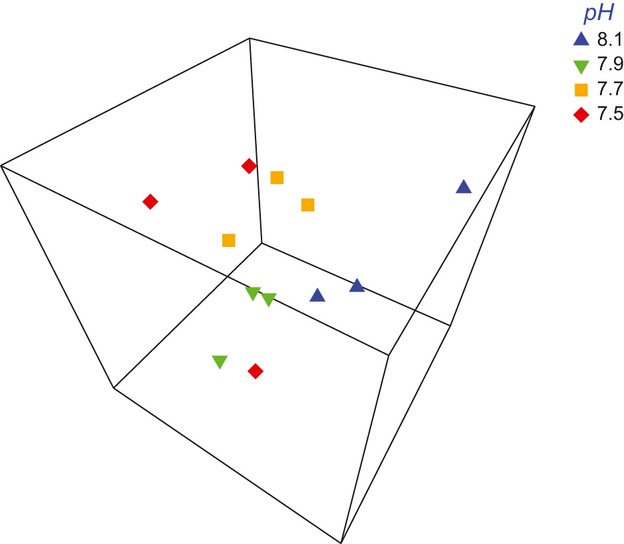
NMDS ordination of OTU (defined at 97% sequence similarity) analysis from triplicate 16S rRNA gene libraries constructed from *Hydrolithon onkodes* exposed to four treatment pH conditions. NMDS is based on a Bray Curtis similarity matrix and the 3D stress value = 0.09.

Unifrac statistical analysis of three independent 16S rRNA gene libraries from each treatment revealed a highly significant difference between all pH treatments (*P* < 0.01) for both weighted and unweighted analyses. This was further visualized in the nMDS ordination of the OTUs (97% sequence similarity) (Fig 3).

No trend according to pH was detected in CCA-derived bacterial diversity and evenness (Table 3, Fig. S2). Rarefaction analysis indicated that between 85 and 95% of the diversity was sampled in the various CCA biofilm treatments (Table 3, Fig. S2). The highest diversity of microbes using both Chao1 and Ace indices was the CCA treated at pH 7.5 and the lowest was CCA treated at pH 7.7.

**Table 3:** Diversity indices calculated from sequences of 16S rRNA genes using a 97% sequence similarity threshold

Treatment	Total clones	OTUs (0.03)	Diversity sampled	Shannon weaver	Chao 1	Ace
pH 7.5	283	98	85	4.05	136	160
pH 7.7	274	73	95	3.78	100	101
pH 7.9	274	85	89	3.85	120	131
pH 8.1	282	81	94	3.94	118	122

**Figure 4:**
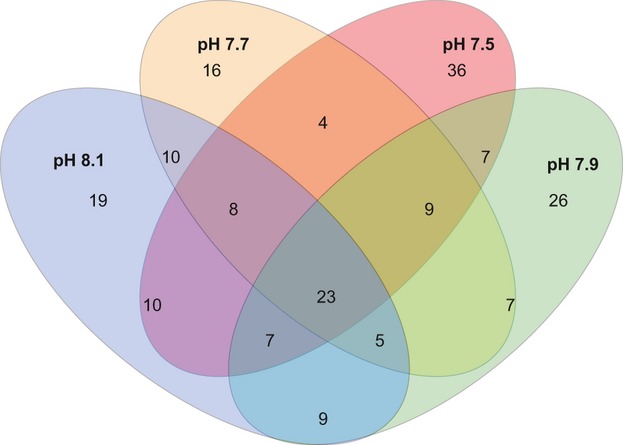
Venn diagram illustrating the number of unique and shared OTUs (97% sequence similarity) within the combined crustose coralline algae derived clone libraries for each pH treatment.

A Venn diagram was constructed to determine how many OTUs were shared across each of the treatments revealing that only 23 OTUs were common to all libraries (Fig 4). The highest number of OTUs detected in a single treatment was 36 OTUs exclusively present on CCA at pH 7.5. The highest (8.1) and lowest (7.5) pH treatments shared 48 OTUs (Fig 4) and 38 OTUs present in CCA at pH 8.1 were lost in CCA treated at pH 7.5.

**Figure 5:**
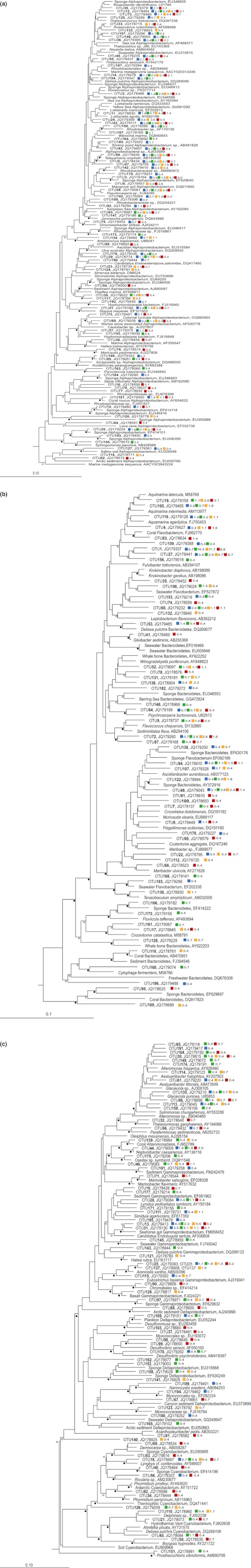
16S rRNA gene-based phylogeny of representative OTUs (at a 97% sequence similarity) within the (a) *Alphaproteobacteria,* (b) *Bacteroidetes* and (c) *Gammaproteobacteria, Deltaproteobacteria* and *Cyanobacteria* displayed in maximum likelihood trees. The relative percentage of each OTU within combined libraries per treatment are indicated alongside each accession number with pH 8.1 in blue, pH 7.9 in green, pH 7.7 in orange and pH 7.5 in red. Filled circles indicate bootstrap support of >90% and open circles represent >50% support. Scale = 10% sequence divergence.

To ascertain the phylogenetic affiliation of each OTU and to determine their relative abundance across treatments, neighbour-joining phylogenetic trees were constructed and the relative percentage each OTU contributed to the combined libraries was added to the tree branches. The microbial communities of CCA from all treatments were dominated by *Alpha* and *Gammaproteobacteria* and *Bacteroidetes* (Fig. S3). Specific OTUs that showed an increased abundance at pH 7.5 relative to pH 8.1 included OTU 3 (15-fold) and OTU 2 (threefold) both within the *Alphaproteobacteria* and most closely related to sequences retrieved from marine sponges (Fig. 5a). The increased abundance of OTU 3 occurred consistently across all pH treatments with fourfold, eightfold, and 15-fold increases detected at pH 7.9, 7.7, and 7.5 respectively. OTUs that showed a decreased relative abundance at pH 7.5 included OTU 94 (fourfold, most closely related to a sponge-associated *Flavobacteria*), OTU 49 (fourfold, most closely related to an *Alphaproteobacteria* sequence retrieved from an electroactive biofilm), 59 (fivefold, most closely related to a gastropod-associated *Alphaproteobacteria)* and the two closely related OTUs 24 and 43 (eightfold and fourfold, respectively, most closely related to *Loktanella agnita*) (Fig 5a and b). Small clusters of OTUs exclusively detected in the 7.5 treatment were also seen within the *Alphaproteobacteria* (OTUs 16, 70 and 77 most closely related to a sequence retrieved from the egg capsules of the squid *Sepia officinalis*) and *Deltaproteobacteria* (OTUs 103, 37, 98, and 99 most closely related to *Desulfovibrio senezil* and *Myxococcales* sp.) although the relative abundance of these within the total treatment library was minor (Fig 5a and c).

## Discussion

The settlement and metamorphosis of coral larvae on CCA was reduced following a 6-week exposure of the algae to seawater adjusted to pH 7.7 (*p*CO_2_ = 1187 μatm) and below. In contrast, there was no acute effect of OA (pH 7.5, *p*CO_2_ = 1638 μatm) on the settlement response of larvae to CCA extracts or CCA that had not been exposed to low pH for 6 weeks. These results indicate that the low pH treatments reduced induction of larval settlement in response to CCA by changing the biochemistry of the CCA or its associated microbial communities. Detailed microbial community analysis of the CCA indicated diverse bacterial assemblages that were significantly different between pH 8.1 (*p*CO_2_ = 464 μatm) and pH 7.9 (*p*CO_2_ = 822 μatm), a condition likely to occur before the end of this century. The shift in microbial community composition continued at lower pH/higher *p*CO_2_ treatments. These differences were primarily due to altered abundance of the dominant operational taxonomic units (OTUs) between the different pH treatments and the appearance of new (but rare) OTUs at pH 7.5, increasing the diversity in this treatment. The effect of experimental duration on microbial community composition was not examined in this study although previous research has shown that CCA-derived microbial communities varied with experimental treatment rather than sampling time and microbial profiles of freshly collected CCA largely overlapped with communities from CCA held in aquaria for 14 days (Webster *et al*., 2011). However, additional analysis of CCA from the collection site would be required to confirm that the microbial populations are directly equivalent. These results highlight the sensitive nature of CCA-derived microbial communities and are consistent with previous studies showing microbial shifts occurring with thermal stress along with reductions in the settlement of coral larvae.

The relationship between *p*CO_2_ exposure of CCA and settlement success (Experiment 1) was linear (SOM Fig 1), with a 16.7% reduction in metamorphosis expected relative to control conditions as *p*CO_2_ increases by 400 μatm. The reduction in larval settlement was observed on CCA exposed to low pH for 6 weeks even though the live surfaces of CCA had not retracted, indicating this effect was not due to reduced availability of CCA nor a change in the CCA assemblage as may be the case for mixed algal communities (Jokiel *et al*., 2008; Albright *et al*., 2010; Doropoulos *et al*., 2012). The process of settlement itself was also not affected by severe but acute OA conditions on CCA that had previously been exposed only to ambient seawater conditions for 6 weeks (Experiment 2). This indicates that the OA-related reduction in settlement is not due to an immediate effect on the physiology of the coral larvae and this was further supported by the results of Experiment 3 where larvae of two species were pre-exposed to the different OA conditions for 24 h and then settled successfully in response to CCA extract in the absence of CCA pieces. These results differ from those of (Nakamura *et al*., 2011), who reported reductions in larval settlement of *Acropora digitifera* larvae after only 2 h pre-exposure to seawater at pH 7.6 in an experiment similar to Experiment 3 in the present study. A key difference between studies may be chemical inducers used to trigger settlement; we used a natural extract of CCA although Nakamura and colleagues used a neuropeptide, which is likely to mimic an internal signalling molecule within the larvae. The results for broadcast spawning species in our study support earlier reports that short-term exposure of CCA to low pH does not affect the settlement of larvae from brooding corals. Albright and Langdon demonstrated that *Porites astreoides* larvae were able to settle on “conditioned” limestone tiles (which support microbial biofilms and possibly CCA communities) during a 24 h exposure to *p*CO_2_ = 800 μatm (Albright & Langdon, 2011). Anlauf and colleagues performed similar experiments and found no effect of OA on the settlement of *Porites panamensis* larvae over 10 days at pH 7.8 (*p*CO_2_ = 950 μatm) (Anlauf *et al*., 2011). A recent study by Doropoulos and co-workers described experiments similar to ours using the same species of coral larvae, but mixed CCA assemblages (Doropoulos *et al*., 2012). In that study settlement was reduced on CCA exposed to pH 7.8 (*p*CO_2_ = 807 μatm) for 60 days (similar to Experiment 1 in the present study) and in a separate experiment on mixed CCA assemblages not previously exposed to OA (similar to Experiment 2). Larval settlement success on CCA at reduced pH over an 18 h period in our experiments contrasts with the reduction in settlement success on mixed CCA over 6 days (Doropoulos *et al*., 2012), but these differences may be due to the exposure durations or single species vs. mixed CCA substrata. Although the effects of OA on the dissolution (Diaz-Pulido *et al*., 2012) and ecological function (this study) of abundant reef-building CCA species such as *H. onkodes* are critical to establish and quantify cause-effect relationships, changes in CCA community structure under conditions of OA may also influence its overall function as a preferred substratum for larval settlement (Doropoulos *et al*., 2012). Further work is required to tease apart the effects of OA on the function of mixed CCA communities, which also support complex microbial communities that may influence metamorphosis of invertebrate larvae.

Larvae of many *Acropora* species do not undergo settlement and metamorphosis in the absence of an inducer provided by CCA or microbial biofilms (Heyward & Negri, 1999; Baird & Morse, 2004; Webster *et al*., 2004), but whether the inducer compound(s) is produced by the CCA or its associated microbial community is still not certain. Here we show that OA does not affect communication between CCA (or its associated microbes) and the coral larvae by modifying the chemistry of the signalling molecule or its detection by the larvae (Experiments 2 and 3). Instead, the longer 6-week exposure of CCA to low pH (Experiment 1) may affect synthesis of the chemical inducer by the CCA directly, by affecting microbial production of the compound or by changing the microbial community structure. This study found that pH significantly affected the structure of the CCA-derived microbial communities with numerous sequences within the *Alphaproteobacteria* and *Bacteroidetes* detected less frequently at pH 7.5 compared to pH 8.1 and novel *Proteobacteria* appearing in the pH 7.5 treatment.

Although individual microbial OTUs increased/decreased abundance at different pHs/*p*CO_2_ and 38 OTUs detected at pH 8.1 were not observed at pH 7.5, none of the abundant OTUs from CCA at pH 8.1 were lost from CCA at pH 7.5. Without knowing the origin and activity of the coral morphogens it is difficult to predict whether or not the reduced abundance of a particular microbe or the loss of a rare species would be sufficient to alter the overall morphogenic chemistry of the CCA. Reduced seawater pH also increased the diversity of bacteria with CCA exposed to pH 7.5 having the largest number of OTU's that were not detected in any other pH treatment. Whether any of these microbes were inhibitory towards coral metamorphosis is also not known. It has been suggested that inhibition of larval settlement in marine invertebrates is primarily based on negative cues (which are extremely common in biofilms and seawater) rather than on the absence of positive cues (Woodin, 1991; Holmström & Kjelleberg, 1994). Many of the microbes reported to inhibit settlement of marine invertebrates reside within the *Gammaproteobacteria* (*Vibrio, Aeromonas, Pseudomonas, Deleya*) (Maki *et al*., 1988; Holmström *et al*., 1992; Mary *et al*., 1993). On CCA there were numerous minor OTUs within the *Gammaproteobacteria* (32, 71, 10, 90) that were exclusive to the pH 7.5 treatment. However, assigning a functional role for these microbes is difficult as they did not cluster with known inhibitory strains and they were absent from the pH 7.7 treatment where a significant reduction in larval settlement was also observed. Targeted cultivation of these microbes in future studies may elucidate their role in larval inhibition. A *Pseudoalteromonas* strain previously isolated from the surface of *H. onkodes* and reported to induce metamorphosis of *A. willisae* larvae (Negri *et al*., 2001) was not detected in any of the OA treatments in the present study.

A shift in the microbial community of CCA occurs in response to both OA (present study) and thermal stress (Webster *et al*., 2011). In the present study one of the most obvious changes was a 15-fold increase in an *Alphaproteobacteria* OTU at pH 7.5 relative to pH 8.1. This OTU appears to thrive under reduced pH conditions with gradual increases detected under each of the declining pH treatments. This is in contrast to changes occurring in response to elevated seawater temperature where a large reduction in *Alphaproteobacteria* and an increase in *Bacteroidetes* was observed. The increased abundance of *Deltaproteobacteria* reported in association with thermal stress was also not observed at low pH, although a cluster of rare *Deltaproteobacteria* sequences exclusive to CCA at pH 7.5 was detected. Similar to the temperature study, these new *Deltaproteobacteria* clustered with *Desulfovibrio* spp., which have been associated with diseased corals and sponges (Webster *et al*., 2008). Under elevated seawater temperatures (32 °C), CCA exhibited bleaching and a reduction in photosynthetic efficiency (*F*_v_/*F*_m_), whereas no visible signs of host stress were detected in the current study at pH 7.5. The difference in the microbial shifts between these two studies may therefore relate to either the individual climate change variables differentially influencing microbial community structure or that the large phyla-level shift observed at elevated seawater temperature may have been linked to the host (CCA) stress response.

CCA and other calcifying algae play critical roles in building reefs and forming structural connections between organisms and reef substrata (Littler & Littler, 1984). The loss of reef-building CCA through OA related reduced calcification or dissolution presents a major risk of habitat loss in both tropical (Anthony *et al*., 2008; Diaz-Pulido *et al*., 2012) and temperate (Russell *et al*., 2009) waters. Studies around shallow water CO_2_ vents indicate that tropical reefs cannot develop where pH is less than 7.7 and *p*CO_2_ is greater than 1000 μatm (Fabricius *et al*., 2011). The importance of CCA to other marine invertebrates as a preferred settlement substratum can also not be understated (Morse *et al*., 1996; Harrington *et al*., 2004), but over 20 years of research has failed to identify the specific chemical or bacterial component responsible for this morphogenic activity towards the larvae of coral or any other marine invertebrate (Tebben *et al*., 2011). Therefore, while OA causes clear shifts in CCA-associated microbes and a negative impact on the ability of CCA to induce metamorphosis in coral larvae, the specific functional component affected by OA remains unclear. Studies on the effects of OA on bacteria are vastly underrepresented in the literature and this study is first to examine the impacts of OA on the potential functional roles of microbes on coral reefs. Although further studies are needed to fully appreciate the potential impacts of OA on microbes, the community shifts identified on CCA and associated reductions in the ability of CCA to initiate coral settlement raise serious concerns for reef development, maintenance and recovery under OA conditions projected to occur into the next century (Orr *et al*., 2005).

## Supporting Information

Figure S1 Regression of % metamorphosis of *A. millepora* larvae against *p*CO_2_ (Experiment 1, *n* = 3 tanks).

Figure S2 Diversity of CCA-derived bacterial communities for each of the 4 pH treatments. Rarefaction curves are based on bacterial OTUs at a 97% sequence similarity threshold.

Figure S2 Phyla and class level differences in bacterial biofilm composition on CCA exposed to four pH treatments. Graphs were constructed using the frequency of 16S rRNA sequences belonging to each bacterial group from clone library analysis.

Table S1 Nested ANOVA of arcsine transformed percent metamorphosis, nested in tanks. Significant effects at *P* < 0.05. DF = degrees of freedom, MS = mean square.
