# Supplementary figures and images for: Ocean acidification reduces induction of coral settlement by crustose coralline algae
Source: Glob Chang Biol. 2012 Sep 25;19(1):303–15. doi: 10.1111/gcb.12008 (PMC3597258; doi:10.1111/gcb.12008)

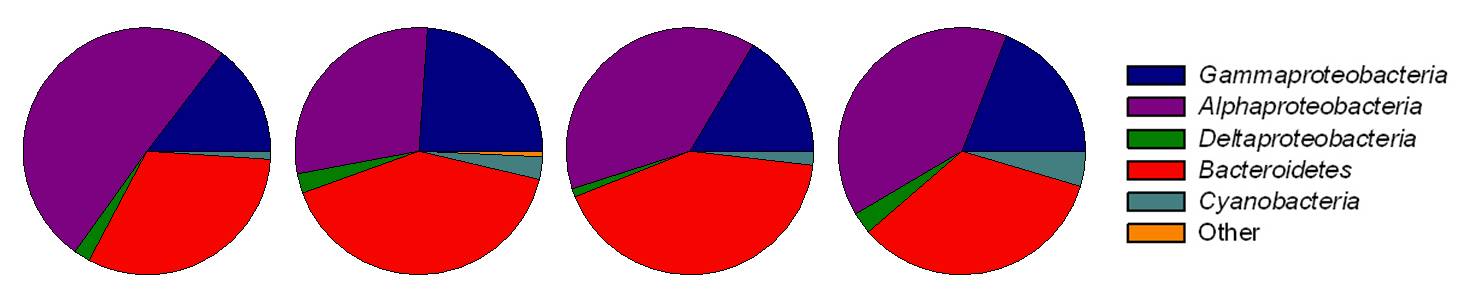

Supplement: Figure S2 — Phyla and class level differences in bacterial biofilm composition on CCA exposed to four pH treatments. Graphs were constructed using the frequency of 16S rRNA sequences belonging to each bacterial group from clone library analysis. [file gcb0019-0303-sd3.jpg]
