# Supplementary material for: Ocean acidification reduces induction of coral settlement by crustose coralline algae
Source: Glob Chang Biol. 2012 Sep 25;19(1):303–15. doi: 10.1111/gcb.12008 (PMC3597258; doi:10.1111/gcb.12008)
Supplement: Table S1 — Nested ANOVA of arcsine transformed percent metamorphosis, nested in tanks. Significant effects at P < 0.05. DF = degrees of freedom, MS = mean square. [file gcb0019-0303-sd4.docx]

Table S1 Nested ANOVA of arcsine transformed percent metamorphosis, nested in tanks. Significant effects at *p* < 0.05. DF = degrees of freedom, MS = mean square.

| Term | DF | MS | F-ratio | Probability |
| --- | --- | --- | --- | --- |
| pH | 3 | 0.1498 | 8.46 | 0.007 |
| Tank | 8 | 1.770 | 0.31 | 0.96 |
| Residuals | 71 | 5.663 |  |  |
